# PHYSICAL SYMPTOMS REACTIVITY TO DAILY STRESSORS AND CARDIOVASCULAR DISEASE: A COMPARISON OF BLACK AND WHITE ADULTS

**DOI:** 10.1093/geroni/igad104.2539

**Published:** 2023-12-21

**Authors:** Sebastiana Moore, Claire Smith, Christina Mu, Soomi Lee

**Affiliations:** University of South Florida, Tampa, Florida, United States; University of South Florida, Tampa, Florida, United States; University of South Florida, Tampa, Florida, United States; The Pennsylvania State University, University Park, Pennsylvania, United States

## Abstract

Black adults have higher risk for cardiovascular disease (CVD) compared to their White counterparts. Biopsychosocial frameworks suggest that this racial difference may not only be due to higher stressor exposure (e.g., discrimination) but also to stressor reactivity (i.e., heightened automatic reactions to stressors). This study examined if racial disparities in CVD is explained by elevated physical symptoms in response to daily stressors (“physical symptom reactivity to stressors”). Data came from 1,727 adults (non-Hispanic Black=38%, non-Hispanic White=62%) who participated in the Midlife in the United States Study at Time-1 (T1: 2004-2009). A subset (n=1,482) was reassessed at Time-2 (T2: 2013-2019). Race, daily physical symptoms, and daily stressors were measured at T1. Physical symptom reactivity to stressors (i.e., individual slope of physical symptoms as a function of daily stressor severity) was calculated using 8-day diary data. Physician-diagnosed CVD was captured at T1 and T2. Indirect pathways (A path: race->stressor physical reactivity, B path: race->CVD) were tested, adjusting for health and sociodemographic covariates. At T1, Black adults exhibited a higher risk of CVD compared to White individuals through higher physical symptom reactivity to stressors (A path: B=0.004, SE=0.0006, p<.001; B path: B=-3.56, SE=1.32, p=.007; indirect effect=0.12, SE=0.03, 95% CI=[0.06, 0.18]). These indirect pathways were also supported when predicting the risk of CVD at T2 after controlling for T1 CVD (B path: B=-3.23, SE=1.56, p=.038; indirect effect=0.07, SE=0.04, 95% CI=[0.02, 0.16]). Findings newly suggest that racial differences in physical manifestations of stress may contribute to disproportionate development of CVD, the deadliest disease in America.

